# A New Operation for Lacerated Perinæum

**Published:** 1855-10

**Authors:** 


*A New Operation for Lacerated Perinæum.* By M. JOBERT.

The peculiarity of this operation is in the suture. After having pared the edges of the wound, M. Jobert threads a broad lace lengthwise through them, and then drawing the lace, he puckers all the edges to a point, precisely as the mouth of a tobacco-pouch is closed by drawing the string. M. Jobert appears to have operated in this way in more than one instance.

CASE. Eliza Dorvilliers, æt. 23, staymaker. The labor, in which the perinæum gave way, was her first and only one; it occurred on April 5th, 1854. The child was dead. Except so far as concerned the wretchedness of the local accident, her recovery was prompt and satisfactory.

She was admitted into the Hopital de la Clinique, under M. Jobert, on the 25th of August, 1854. At this time her general health was pretty good. Three days previously she had menstruated for the first time since her confinement. She had no power of retaining flatus, or fæces, if at all fluid. The perinæum is completely lacerated, and the recto-vaginal septum is torn to a certain extent (about 3 centimetres). The lacerated edges are retracted and cicatrized. The neighboring skin is red and somewhat tumefied. Having prepared the patient by several baths and a purgative enema, administered the night before, M. Jobert proceeded to operate on the 1st of September.

Having placed the patient in a position for lithotomy, the edges of the wound were pared. This took considerable time, and it was attended with much hemorrhage. Then three threads were taken and introduced in succession, the one through the length of the upper border of the wound, the second through one side of the lacerated perinæum, and the third through the other side. After this, these threads were drawn tight and tied in a double knot. Last of all, incisions were made on each side, to take off the tension.

The case progressed favorably without any remarkable event. On the 6th, the threads were removed, and union appeared complete. On the 8th, there having been no motion up to this time, several unwise efforts were made at the close-stool, but without any ill consequences. On the 15th, a careful examination was made, when there was found to be a minute fistulous communication between the vagina and rectum. This was touched with a point of lunar caustic. On the 24th, this opening was completely closed, and the patient had recovered complete power over the bowel. The perinæum is about 3 centimetres broad, and appears to be very solid. On the 26th October, she left the hospital quite well.—*Gaz. Hebd. de Med. Ranking's Abstract.*

